# Aesthetic experiences and flourishing in science: A four-country study

**DOI:** 10.3389/fpsyg.2022.923940

**Published:** 2022-08-09

**Authors:** Christopher J. Jacobi, Peter J. Varga, Brandon Vaidyanathan

**Affiliations:** ^1^Department of Sociology, Nuffield College, University of Oxford, Oxford, United Kingdom; ^2^Department of Experimental Psychology, Christ Church, University of Oxford, Oxford, United Kingdom; ^3^Department of Sociology, The Catholic University of America, Washington, DC, United States

**Keywords:** aesthetics, beauty, awe, wonder, flourishing, scientific work, mental health crisis

## Abstract

In response to the mental health crisis in science, and amid concerns about the detrimental effects of the COVID-19 pandemic on scientists, this study seeks to identify the role of a heretofore under-researched factor for flourishing and eudaimonia: aesthetic experiences in scientific work. The main research question that this study addresses is: To what extent is the frequency of encountering aesthetics in terms of beauty, awe, and wonder in scientific work associated with greater well-being among scientists? Based on a large-scale (*N* = 3,061) and representative international survey of scientists (biologists and physicists) in four countries (India, Italy, the United Kingdom, and the United States), this study employs sets of nested regressions to model the associations of aesthetic experiences with flourishing while controlling for demographic factors and negative workplace and life circumstances such as burnout, job/publication pressure, mistreatment, COVID-19 impacts, other stressful life events, serious psychological distress, and chronic health conditions. The results show that the frequency of aesthetic experiences in scientific work in the disciplines of biology and physics has a very large and statistically significant association with flourishing and eudaimonia that remains robust even when controlling for demographic factors and negative workplace and life circumstances, including COVID-19 impacts. Aesthetic experiences in scientific work are even as strongly associated with flourishing as the presence of serious psychological distress and are most strongly associated with the flourishing domain of meaning in life, thus pointing to a link with eudaimonic well-being. In line with neurophysiological evidence and positive psychological models of flow, self-transcendence, and intrinsic motivation, aesthetics are a key source of flourishing for scientists in the disciplines of biology and physics. While future research needs to test the causal mechanism, the strength of the findings could encourage leaders of scientific labs and research organizations generally to remove obstacles to experiencing the aesthetic dimensions of science. Fostering cultures in which the aesthetic experiences that are intrinsic to scientific practice are fully appreciated might potentially protect or boost flourishing by reducing the impacts of burnout, job/publication pressure, and mistreatment-related experiences in science.

## Introduction

Recent research has drawn attention to what appears to be a growing mental health crisis among scientists and in academia more generally (Eisenberg et al., 2007; Gewin, 2012; Garcia-Williams et al., 2014; Evans et al., 2018; Bleasdale, 2019). The pressurized climate created in research institutions has been found to be particularly detrimental to scientists (de Meis et al., 2003). The emphasis on productivity in the selection of graduate students and the hiring of new faculty makes the problem more acute (Duffy et al., 2011). These effects are compounded by high levels of bullying and harassment that appear to be entrenched in existing research group power dynamics and organizational cultures (Keashly, 2015; Moss, 2018; Bleasdale, 2019). Correspondingly, rates of burnout among academics and researchers have reached levels experienced by workers in high-risk environments (Watts and Robertson, 2011; Bleasdale, 2019).

Burnout and attrition in science, particularly stemming from unhealthy organizational cultures, has been documented in both academic and industry reports (de Meis et al., 2003; Duffy et al., 2011; HR Research Institute, 2019; Brower, 2022). Moreover, the pressure to publish for continued career progress and funding has added an unhealthy competitive edge to the research culture in many places, leading some to suggest that scientometrics have come to be valued more than real knowledge and that the mental health of the researchers is the price paid to offset the shortcomings of vital resources and funding (de Meis et al., 2003; Peterson et al., 2008). In the bleakest projections of this reality, some sociologists and psychologists warn that not only will current scientific professionals experience adverse effects, such as stress and burnout, but also future generations will be discouraged from embarking on scientific careers as a result (de Meis et al., 2003; Duffy et al., 2011). While identifying the prevalence of negative outcomes is an important diagnostic, in order to address this crisis of well-being in science—which has likely been exacerbated by the COVID-19 pandemic (Chan et al., 2020), more research is needed on what contributes to scientists’ flourishing as well.

Aesthetic experiences are one such potential source of well-being that has yet to be examined on a large systematic scale. Scientists as well as philosophers of science typically conceptualize aesthetics in scientific workplaces in terms of experiences of beauty (e.g., symmetry, elegance), wonder, and awe (McAllister, 1996; Dawkins, 2000; Wilczek, 2016; Gilbert, 2018; Gottlieb et al., 2018; Hossenfelder, 2018; MacArthur, 2021). Relatedly, experiences of beauty, awe, and wonder are identified in the psychological literature and tradition as definitive of aesthetics (Scarry, 1999; Hagman, 2002; Keltner and Haidt, 2003; Feynman, 2007; Shiota et al., 2007; Valdesolo and Graham, 2014; Darbor et al., 2016; Nakayama et al., 2020). They also, respectively, cover the three neural systems implicated in the neuroaesthetic triad—sensory-motor, knowledge-meaning, emotion-valuation (Chatterjee and Vartanian, 2016)—as well as the three primary domains of psychological interest relevant to self-transcendence—motivation, cognition, emotion (Marković, 2012; Varga, 2021). The connection between science and aesthetics thus has both philosophical roots (Breitenbach, 2013; Montano, 2013; Arcangeli and Dokic, 2020) and neuropsychological underpinnings (e.g., Zeki et al., 2014; Gottlieb et al., 2018). Aesthetic experiences are proposed to play an important role in scientists’ lives and the field more broadly, from fostering education and facilitating communication (Girod et al., 2003, 2010; Girod, 2007; Ivanova, 2017) to motivating research and shaping theory (McAllister, 1996; McLeish, 2019; MacArthur, 2021).

Self-transcendent aesthetic experiences, in particular, are theorized to be inherently positive as narrowly defined (Pak, 2019; Thrash, 2021) and thus offer a vital avenue to better understanding how scientists can thrive today (Owens, 2022). Thriving—a central component of and nearly synonymous with eudaimonic well-being—has itself been examined in a variety of different ways (Ryan and Deci, 2001; Su et al., 2014; Belzak et al., 2017). Drawing on the ancient Greek philosophical tradition (e.g., Aristotle, 2001), well-being researchers and positive psychologists have increasingly gravitated towards measuring thriving in terms of human flourishing, which includes aspects of well-being (e.g., meaning, virtue, character, relationships) which are less commonly accounted for by other well-being constructs (VanderWeele, 2017). Aesthetic experiences in scientific work are hypothesized to contribute to this form of thriving in scientists.

Advances in neuroscientific and psychological research, particularly in the burgeoning field of neuroaesthetics, support a link between aesthetic experiences and well-being (see Mastandrea et al., 2019 for a review). Moreover, neuroimaging has revealed the same cortical response patterns to abstract mathematical beauty as visual artistic beauty, suggesting the underlying neuroscientific link between ordinary aesthetic experience and positive mental health outcomes can be extended to analogous positive effects from scientific aesthetic experiences (Kawabata and Zeki, 2004; Jacobs et al., 2012; Zeki et al., 2014). This research has heretofore largely focused on establishing a relationship between aesthetic apperception and affective pleasure (i.e., hedonic well-being; Berridge and Kringelbach, 2015). For example, aesthetic objects are often considered in terms of eliciting pleasant feelings, and aesthetic emotions are often categorized within the realm of pleasure (Keltner and Haidt, 2003), while a variety of neuroscientific studies of aesthetic experiences have focused on neural regions associated with reward such as the nucleus accumbens, amygdala, and ventral tegmentum (Jacobs et al., 2012).

It is, however, important to also consider the modality (e.g., mundane or hedonic versus transmissive or transcendent) of the aesthetic experience in addition to its content. When aesthetic contents are experienced in characteristically transcendent ways, the effect is qualitatively different from more mundane experiences of prettiness or pleasantness and closely parallels other higher states (Marković, 2012), indicating a largely underexplored relationship between aesthetics and well-being. The present research thus responds to calls for increased attention to eudaimonic and self-transcendent well-being (Huta and Ryan, 2010; Belzak et al., 2017; Thrash, 2021) and establishes a relationship between aesthetic experiences and scientists’ well-being, as indicated by human flourishing.

Positive psychologists have sometimes considered pathways between aesthetic experiences and flourishing. For instance, the well-being benefits of flow experiences (i.e., the state when a person becomes fully immersed in an activity like playing an instrument; Nakamura and Csikszentmihalyi, 2014) have been linked to aesthetic experiences and arts education (Wanzer et al., 2020). Aesthetic experiences could mirror theoretical models of positive emotions that “broaden and build” awareness and psychological resources over time (Fredrickson, 2004) and thereby lead to higher well-being, pathways that have been found to have neurophysiological evidence (Garland et al., 2010). One could also conceive of aesthetics as important for self-actualization and realization (Heintzelman, 2018), especially if scientists were motivated by beauty and wonder when they chose their career path or when they felt a sense of vocation to a scientific career. In the broadest sense, this study contributes to positive psychology by explicitly exploring the relevance of aesthetics as positive factors for flourishing (Seligman, 2002).

By collecting a large international dataset of scientists in the disciplines of biology and physics and employing sophisticated population weights, we address the sampling limitations and overestimation concerns raised in response to previous studies of mental health in academia (Monlong, 2018). With this greater precision and in line with the above literature, we advanced three hypotheses. First, the frequency of aesthetic experiences in scientific work would be positively associated with overall human flourishing. Second, the association of aesthetic experiences in scientific work with flourishing would remain significant even when accounting for the negative aspects of working in science (burnout, job/publication pressure, and mistreatment) as well as when controlling for general stressors (COVID-19, other stressful life events, chronic health conditions, and serious psychological distress) that might impact flourishing. Third, the link between aesthetics and flourishing would be most pertinent for the eudaimonic domain of meaning in life from the overall flourishing measure. As discussed, scientists could find higher aesthetic experiences to be inherently meaningful as opposed to just merely pleasant and those experiences could lead to eudaimonic flourishing. The flourishing index used is a multi-dimensional construct of several domains (life satisfaction, physical health, mental health, meaning in life, character, close social relationships, and financial stability), which both captures overall flourishing and allows for detailed analysis of the different dimensions.

## Methods

### Participants

Data for this study come from an international survey of scientists in physics and biology departments at PhD granting institutions and research institutes in Italy, India, the United Kingdom, and the United States. We selected physics and biology in order to focus on two core scientific disciplines which are recognized to have distinct approaches to aesthetics (MacArthur, 2021). While we cannot generalize from these disciplines to the entire scientific community, examining these disciplines provides insights into distinct types of aesthetic judgments in scientific practice.

The four countries were selected for a number of reasons. First, we wanted to examine aesthetics and well-being in countries with distinctive societal contexts and differences in scientific infrastructure. Given the different geographical locations in which the countries are located (i.e., in North America, West Europe, and South Asia, respectively), we expected that differences in their social contexts might influence how scientists in these countries experience and express emotions and the extent to which they are attuned to aesthetic considerations. The four countries also differ in their scientific infrastructures: measured by the percentage of GDP spent on research and development (R&D), the United States has the most developed scientific infrastructure among the four countries (2.83%) followed by the United Kingdom (1.7%), Italy (1.39%), and India (0.65%; The World Bank, 2021). The four countries also have distinctive cultural histories which likely shape aesthetic traditions and formation, which in turn might affect the aesthetic experiences of scientists in those countries. Finally, the four countries were chosen because they garnered the highest survey response rates in a previous international survey of scientists conducted by one of the authors (Ecklund et al., 2019), and we already had networks of research collaborators in place to facilitate data collection in these countries, ensuring a higher probability of success in carrying out the study than if we had chosen other countries.

The survey was nationally representative of the target population in each country (starting *N* = 3,442). Data collection occurred in two waves: from May to June 2021 and from August to October 2021; a detailed methodological report and other study materials are available in the public repository of this study (Vaidyanathan and Jacobi, 2022). These two time frames co-occurred with the dynamic COVID-19 pandemic situations in the four countries (e.g., Indian coronavirus infection rates peaked during the first survey wave^1^). Respondents received a $20 (USD or country-specific equivalent) e-gift card and took the survey in English (apart from scientists in Italy, who had the option of taking the survey in Italian or English). Levels of missingness were low: 381 of 3,442 respondents were dropped because they had a missing response on at least one of the below variables, resulting in a final sample of 3,061 respondents. This study received human subjects research approval from the Institutional Review Board of The Catholic University of America (21-0005). Informed consent was obtained from all participants.

### Aesthetic experiences in scientific work

**Figure 1:**
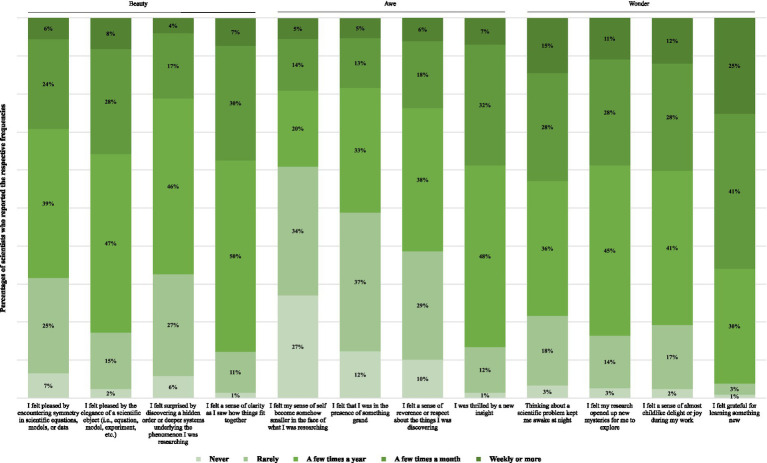
Frequencies of 12 types of aesthetic experiences in scientific work (*N* = 3,061).

We included 12 indicators on the frequency of aesthetic experiences in scientific work (Figure 1). The Likert scales were anchored at 0 = *never*, 1 = *rarely*, 2 = *a few times a year*, 3 = *a few times a month*, to 4 = *weekly or more*. Although exploratory, the items were meant to capture the three domains of beauty, awe, and wonder that are dominant in the extant literature on aesthetics. The items for awe align closely with the well-established Awe Experience Scale (AWE-S; Yaden et al., 2019)—an awe item related to experiencing a sense of vastness, for instance: “I felt that I was in the presence of something grand.” Based on a review of literature, consultations with academic experts in the field of the psychology of emotion, and in-depth qualitative interviews with scientists in the four countries between 2012 and 2019, original items on beauty (e.g., symmetry and elegance) and wonder (e.g., curiosity) were developed. These state items follow the well-established dispositional positive emotion scale (DPES) in combining beauty, awe, and wonder (Shiota et al., 2006) to form an integrated measure of aesthetic experiences.

**Figure 2:**
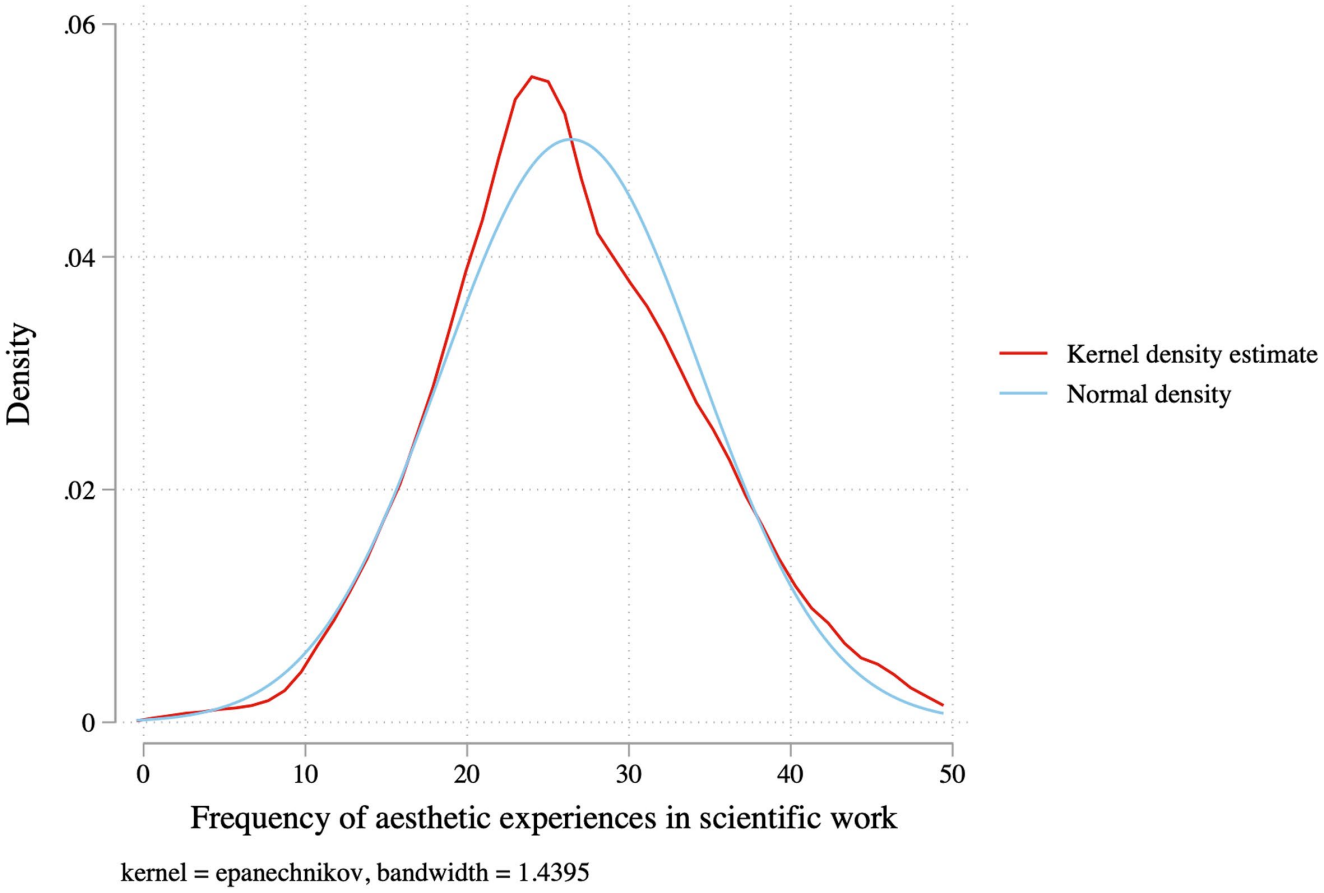
Kernel density plot of the frequency of aesthetic experiences in scientific work (*N* = 3,061).

Exploratory factor analyses revealed that the 12 frequency items load on a single underlying latent factor of aesthetic experience that encompasses beauty, awe, and wonder (Supplementary material 1; the first factor has an eigenvalue of 5.05 and a second factor would only have an eigenvalue of 0.49), and the alpha score of all items is favorable at 0.89. Because of the greater interpretability of a summated frequency scale and because we had no *a priori* assumption about the relative importance of the items (Allum, 2015), however, a summative scale of the frequency of aesthetic experiences in scientific work is used with a potential range of 0–48 (i.e., from 0 or never having any of the 12 aesthetic experiences to 48 or experiencing all of them at a frequency of weekly or more). Furthermore, this summative scaling method also provides parity with how the outcome variable of flourishing is measured. The distribution of the frequency of aesthetic experiences scale roughly resembles the shape of a normal distribution (Figure 2). While some aesthetics items could seem to be hedonic (e.g., “I felt pleased by the elegance of a scientific object (i.e., equation, model, experiment, etc.)”) and others more eudaimonic (e.g., “I felt grateful for learning something new”), those aspects could be related, or scientists could potentially experience them in a transcendent way. This exploratory study does not further distinguish the nature of the aesthetic experiences.

### Negative workplace and life circumstances

We include an indicator for the hallmark emotional exhaustion domain of burnout (“I feel emotionally exhausted whenever I think about my work”), measured on a 5-point Likert scale from 1 = *completely disagree* to 5 = *completely agree*. This item closely mirrors the measure from the Maslach Burnout Inventory (Maslach et al., 1997; Maslach and Leiter, 2021). On the same response scale, we measure feelings of job/publication pressure (“I constantly feel pressure to publish or win grant funding”). Furthermore, mistreatment in the scientific career is a binary measure of any “yes” responses to ever having experienced any of the following types of mistreatment: “harassment,” “public humiliation or shaming,” “bullying,” “verbal abuse,” “discrimination,” and “malicious gossip or rumors.”^2^

Given the timing of the survey during the COVID-19 pandemic, we included two binary indicators, one for having personally been infected by SARS-CoV-2 (“I was infected with COVID-19”) and another for close interpersonal impact of the virus (“someone close to me passed away or became seriously ill during the pandemic”) where participants were asked to respond as applicable from a list of statements. Responses were coded as 1 if the respondents selected “yes” and 0 otherwise. We further included a binary indicator for other stressful life events: “other than the pandemic, in the past 12 months, have you experienced any stressful life-events (e.g., serious injury, serious financial strain, divorce, death of a loved one, etc.)?” and any chronic health or mental health conditions that a survey respondent may have had: “are you being treated for, or do you have a diagnosis for, any chronic physical or mental health condition (e.g., cancer, heart disease, anxiety disorder, bipolar disorder, etc.)?” We additionally included a binary indicator for self-reported serious psychological distress which was coded as 1 if respondents had a score of 13 or higher on the Kessler K6 scale (the K6 consists of six mental illness items such as “hopelessness” or “nervousness” over the last 30 days which were each scored from 0 = *never* to 4 = *all of the time*, resulting in a total range of 0–24; Kessler et al., 2002).

### Demographic control variables

We controlled for country (India, Italy, the United Kingdom, and the United States), position/status (postgraduate student, postdoc, research scientist, junior faculty, mid-level faculty, senior faculty, categorical specification), discipline (physics, biology, other), age (continuous), gender (men scientists, women scientists), the number of children 18 years of age and under currently living in the household (from 0 = *no children*, 1 = *one child*, 2 = *two children*, 3 = *three or more children*; treated as continuous), and survey wave (May to June 2021, August to October 2021; capturing differences in the pandemic-related situations between the countries) in all models.

### Dependent variables

Our key outcome variable of flourishing is measured on a reduced format of the Flourishing Index (VanderWeele et al., 2020). This comprehensive index is validated for the assessment of complete human well-being (flourishing) and is designed for the promotion of health and well-being as opposed to focusing on health deficiencies (Weziak-Bialowolska et al., 2021). The meaning in life and character (albeit to a lesser extent) domains capture conceptions of eudaimonia most closely (Lee et al., 2021). The items themselves were taken from other well-established measures and the item on meaning in life is one of the most common measures of eudaimonia in cross-national research (OECD, 2020).

The full scale of the flourishing index includes two items for each domain, but because of space constraints on the survey, a reduced set was employed, encompassing all dimensions of flourishing that were mentioned in the previous section (life satisfaction: “Overall, how satisfied are you with life as a whole these days?,” physical health: “In general, how would you rate your physical health?,” mental health: “How would you rate your overall mental health?,” meaning: “Overall, to what extent do you feel the things you do in your life are worthwhile?,” character: “I always act to promote good in all circumstances, even in difficult and challenging situations,” close social relationships: “I am content with my friendships and relationships,” and financial stability: “How often do you worry about being able to meet normal monthly living expenses?”).^3^ A response of 0 represents the worst possible and a response of 10 represents the maximum possible score on each domain; for the overall flourishing index all domains are summated for a potential range of 0 (worst possible) to 70 (best possible).

### Statistical models

We used ordinary least squares (OLS) regression techniques to model associations of aesthetic experiences with flourishing *via* two nested models to assess the relative contributions of aesthetic experience frequency on flourishing (Hypotheses 1 and 2). In model 1 (M1) we induce the frequency of aesthetic experience scale with the demographic and socioeconomic control variables. In model 2 (M2) we add the negative life circumstances, which encompass workplace and life stressors as this allows us to control for burnout, negative mental health and other health problems which are likely to influence flourishing. The literature suggested that the associations of aesthetics should be particularly pertinent for the most eudaimonic domains of flourishing (Hypothesis 3). Accordingly, in models 3 (M3a-g), we run OLS models with the frequency of aesthetic experiences and the baseline control variables separately for each domain of flourishing. All analyses, including the descriptive statistics, have been survey weighted.

## Results

Descriptive statistics are presented in Table 1. The mean score of flourishing across all respondents was moderately high at 50.5. 66% of scientists in the disciplines of biology and physics reported a stressful life environment during the last 12 month of the pandemic. Reports of mistreatment (55% having experienced at least one form of mistreatment), job/publication pressure (53% mostly or completely agreeing on the Likert scale), and burnout (23% mostly or completely agreeing) were also elevated. A total of 20% of scientists experienced someone close pass away or become seriously ill during the pandemic, 17% had a chronic health condition, 13% could be classified as having serious psychological distress, and 6% were infected with COVID-19 at any point during the pandemic.

**Table 1:** Descriptive statistics (*N* = 3,061).

	Svy. proportion	Svy. mean	Minimum	Maximum
Flourishing		50.55	5.00	70.00
Frequency of aesthetic experiences in scientific work		25.31	0.00	48.00
Negative workplace and life circumstances				
Burnout: emotional exhaustion domain		2.44	1.00	5.00
Job/publication pressure		3.39	1.00	5.00
Mistreatment in the scientist’s career	0.46			
Having been infected by COVID-19 during the pandemic	0.06			
Someone close passed away or became seriously ill during the pandemic	0.20			
Other personal stressful life events during the last 12 months	0.66			
Serious psychological distress (K6 cut-off scoring)	0.13			
Chronic health condition	0.17			
*Country*				
United States	0.55			
United Kingdom	0.27			
India	0.10			
Italy	0.09			
*Discipline*				
Physics	0.52			
Biology	0.38			
Other	0.10			
*Position/status*				
Postgraduate student	0.30			
Postdoc	0.16			
Research scientist	0.05			
Junior faculty	0.12			
Mid-level faculty	0.11			
Senior faculty	0.27			
Gender: women scientist (ref. men scientist)	0.32			
Age		42.23	18.00	86.00
Number of kids		1.53	1.00	4.00
Survey wave: August–October 2021 (ref. May–June 2021)	0.68			

Work and Well-Being in Science: An International Study (2021). Svy., fully survey-weighted statistics. Proportions correspond to binary or categorical variables; means to variables that are continuous or to Likert-scales that are treated as continuous.

The mean score for frequency of aesthetic experiences in scientific work was 25.3. Looking at the individual aesthetics items (Figure 1; the grouping by beauty, awe, and wonder is not strict but only used for ease of interpretability), experiences of wonder are highest on average (e.g., around 40% of scientists reported “I felt a sense of almost childlike delight or joy during my work” at least a few times a month) and over 11% of all scientists reported all four wonder experiences to happen weekly or more often. Experiences of beauty were also frequent (e.g., 88% of scientists reported “I felt a sense of clarity as I saw how things fit together” at least a few times a year, and 31% reported the same a few times a month or more often). Experiences of awe were generally less frequent (e.g., only 39% reported “I felt my sense of self become somehow smaller in the face of what I was researching” at least a few times a year, only 19% reported this at least a few times a month or more often, while the portion who never experienced it was pronounced at 27%). On another awe item, however, 87% of scientists experienced “I was thrilled by a new insight” at least a few times a year. While most scientists report middle frequencies of a few times a year or a few times a month across most aesthetic experiences in scientific work, there is sufficient variation at the most frequent (4–25% for frequencies of weekly or more across the 12 items) and least frequent (1–27%) levels of aesthetic experiences in science.

**Figure 3:**
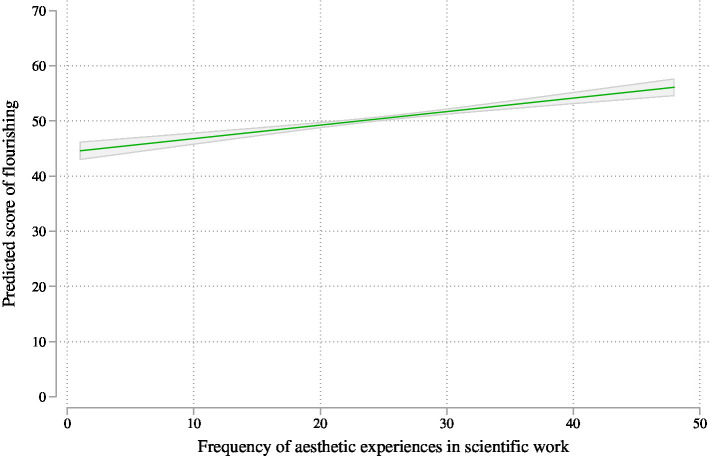
Predicted probabilities of flourishing by the frequency of aesthetic experiences in scientific work (*N* = 3,061).

The Pearson correlation between the frequency of aesthetic experiences and flourishing was *r* = 0.19 (*p* < 0.001). Based on the regression analyses and in relation to the first hypothesis (Table 2), we find that the frequency of aesthetic experiences in scientific work has a highly significant and large association with flourishing (*B* = 0.25, *p* < 0.001) while holding the other control variables constant. As visualized in Figure 3, scientists who never have aesthetic experiences have a lower predicted score of flourishing [44.3 (95% CI: 42.7–46.0)] than those with the highest frequency of aesthetic experiences [55.9 (95% CI: 54.4–57.4)]. This almost 12-point difference in flourishing is considerable, especially as it accounts for the associations of the control variables with flourishing. The amount of variance explained by the variables in this model is high, at 17%. By comparison with the largest associations of the demographic control variables, differences in flourishing by academic position account for up to 4.50 points (e.g., when comparing postgraduate students with mid-level faculty). There were not any statistically significant disciplinary or gender differences in flourishing and the number of children as well as the survey wave did not correspond to differences in flourishing either. The country level differences with the US as the reference group and holding the other variables constant show that on average flourishing was lower in the United Kingdom and India but higher in Italy.

**Table 2:** OLS regression results (models 1 and 2) of flourishing onto the frequency of aesthetic experiences in scientific work.

	M1 Flourishing	M2 Flourishing
	*B* (95% CI)	*B* (95% CI)
Frequency of aesthetic experiences in scientific work^a^	0.25^***^ (0.18–0.31)	0.16^***^ (0.10–0.21)
**Negative workplace and life circumstances**		
Burnout: emotional exhaustion domain^a^		−2.12^***^ (−2.82 to −1.42)
Job/publication pressure^a^		−0.42 (−1.16 to 0.32)
Mistreatment in the scientist’s career		−1.72^**^ (−2.90 to −0.55)
Having been infected by COVID-19 during the pandemic		−1.11 (−2.95 to 0.72)
Someone close passed away or became seriously ill during the pandemic		−0.04 (−1.30 to 1.23)
Other personal stressful life events during the last 12 months		−1.69^***^ (−2.59 to −0.79)
Serious psychological distress (K6 cut-off scoring)		−7.78^***^ (−8.54 to −7.03)
Chronic health condition		−1.63^*^ (−3.11 to −0.15)
**Demographic and socioeconomic controls**		
Country: United Kingdom (ref. United States)	−1.28^*^ (−2.44 to −0.11)	−0.66+ (−1.44 to 0.12)
Country: India (ref. United States)	−2.42^***^ (−3.45 to −1.40)	−2.18^***^ (−3.09 to −1.26)
Country: Italy (ref. United States)	1.75^***^ (0.99–2.52)	2.03^***^ (1.29–2.77)
Discipline: Biology (ref. physics)	−0.89 (−2.18 to 0.39)	−0.03 (−0.82 to 0.76)
Discipline: Other (ref. physics)	−2.46+ (−5.09 to 0.18)	−1.15 (−3.17 to 0.87)
Position/status: postdoc (ref. postgraduate student)	1.56^**^ (0.45–2.67)	1.06^**^ (0.27–1.86)
Position/status: research scientist (ref. postgraduate student)	2.56^**^ (0.80–4.32)	1.24 (−0.85 to 3.33)
Position/status: junior faculty (ref. postgraduate student)	2.91 (−0.76 to 6.58)	2.00 (−1.21 to 5.20)
Position/status: mid-level faculty (ref. postgraduate student)	4.53^**^ (1.75–7.30)	3.12^*^ (0.70–5.54)
Position/status: senior faculty (ref. postgraduate student)	4.30^*^ (0.56–8.05)	2.71+ (−0.38 to 5.81)
Gender: women scientist (ref. men scientist)	−1.05+ (−2.15 to 0.05)	0.32 (−0.83 to 1.46)
Age^a^	0.11^*^ (0.00–0.22)	0.06 (−0.04 to 0.16)
Number of children^a^	0.11 (−0.74 to 0.96)	−0.20 (−0.86 to 0.45)
Survey wave: August–October 2021 (ref. May–June 2021)	−0.01 (−1.43 to 1.41)	0.29 (−0.81 to 1.38)
Observations	3,061	3,061
R-squared	0.17	0.42

Work and Well-Being in Science: An International Study (2021). *B*, unstandardized regression coefficients; CI, confidence interval. The statistics are fully survey-weighted. OLS regression of human flourishing as measured through the Flourishing Index.

a Treated as continuous; otherwise, the variables are categorical or binary.

^***^p < 0.001, ^**^p < 0.01, ^*^p < 0.05, ^+^*p* < 0.1.

In model 2, we considered the negative life stressors as mediators and found that both burnout (*B* = −2.12, *p* < 0.001) and having experienced mistreatment in the scientist’s career (*B* = −1.72, *p* < 0.001) have highly significant negative associations with flourishing. Increasing levels of job/publication pressure do not appear to be negatively associated with flourishing, but some of the potential association might already be explained by burnout. Interestingly, the two COVID-19 variables do not have a statistically significant association with flourishing. Having experienced other stressful life events in the last 12 months during the pandemic is negatively linked with flourishing (*B* = −1.69, *p* < 0.001). As expected, having serious psychological distress is a very strong predictor of flourishing (*B* = −7.78, *p* < 0.001). Yet, the association of aesthetic experiences in scientific work remains robust and large (*B* = 0.16, *p* < 0.001) even if it is slightly diminished (around 36%) when compared to model 1. This suggests that aesthetic experiences in scientific work have a positive and independent association with flourishing even when one accounts for the negative aspects of scientific work, psychological distress or negative life circumstances in general. Nonetheless, only a minor part of this association is mediated *via* these stressors. The difference between the least frequent to most frequent aesthetic experiences in scientific work is as strongly associated with flourishing as having a serious psychological distress. The share of explained variance increases significantly in model 2–42% which is expected given the inclusion of serious psychological distress and other negative life circumstances.

**Figure 4:**
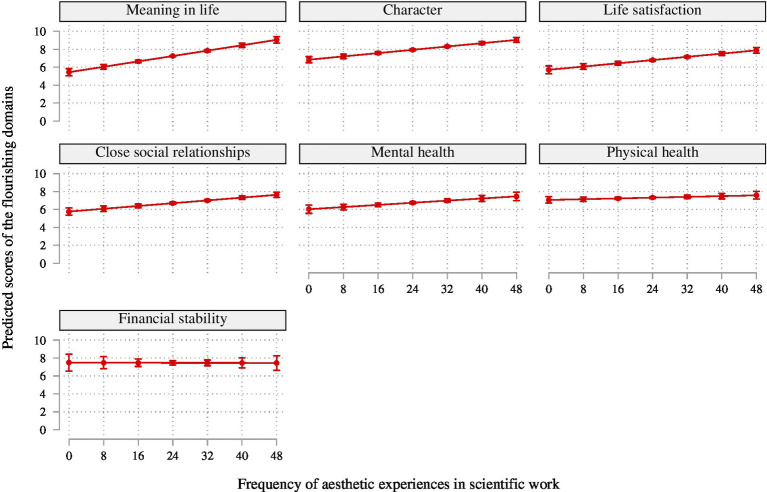
Predicted probabilities of the separate flourishing domains by the frequency of aesthetic experiences in scientific work (*N* = 3,061).

Moving on to models 3a–g to examine the individual dimensions of flourishing (Table 3), the results show that the association of aesthetic experiences in scientific work is strongest for meaning in life (*B* = 0.08, *p* < 0.001), slightly less strong for character and life satisfaction (*B* = 0.05, *p* < 0.001) and close social relationships (*B* = 0.04, *p* < 0.001), and weakest for mental health (*B* = 0.03, *p* < 0.01) domains but insignificant for the physical health and financial stability domains. Similarly, the share of explained variance is highest for the meaning in life domain, at 21%, and lower in the other domains. These different associations indicate that aesthetic experiences are most strongly linked to eudaimonic well-being in terms of meaning in life. The size of the association is very substantive for the meaning domain at almost 3 points on the 11-point scale of the domain (Figure 4).

**Table 3:** OLS regressions (models 3a–g) of the flourishing domains onto the frequency of aesthetic experiences in scientific work.

	M3a Life satisfaction	M3b Physical health	M3c Mental health	M3d Meaning in life	M3e Character	M3f Close social relationships	M3g Financial stability
	*B* (95% CI)	*B* (95% CI)	*B* (95% CI)	*B* (95% CI)	*B* (95% CI)	*B* (95% CI)	*B* (95% CI)
Frequency of aesthetic experiences in scientific work^a^	0.05^***^ (0.03–0.06)	0.01 (−0.01–0.03)	0.03^**^ (0.01–0.05)	0.08^***^ (0.06–0.09)	0.05^***^ (0.03–0.06)	0.04^***^ (0.03–0.05)	−0.00 (−0.04 to 0.03)
**Demographic and socioeconomic controls**							
Country: United Kingdom (ref. United States)	−0.14 (−0.41 to 0.14)	−0.12 (−0.28 to 0.03)	−0.31 (−0.70 to 0.07)	−0.20+ (−0.42 to 0.01)	−0.06 (−0.32 to 0.20)	0.13 (−0.15 to 0.41)	−0.57^*^ (−1.08 to −0.06)
Country: India (ref. United States)	−0.56^***^ (−0.72 - -0.40)	−0.44^***^ (−0.63 - -0.24)	0.08 (−0.18 to 0.34)	−0.37^***^ (−0.53 −0.22)	−0.47^***^ (−0.69 - -0.26)	0.29^**^ (0.09–0.49)	−0.95^***^ (−1.39 to −0.52)
Country: Italy (ref. United States)	0.44^***^ (0.28–0.60)	0.41^***^ (0.25–0.57)	0.82^***^ (0.62–1.02)	−0.03 (−0.15 to 0.09)	0.59^***^ (0.43–0.74)	0.55^***^ (0.36–0.74)	−1.02^***^ (−1.49 to −0.55)
Discipline: Biology (ref. physics)	−0.04 (−0.26 to 0.18)	−0.18 (−0.39 to 0.04)	−0.23 (−0.60 to 0.15)	0.01 (−0.16 to 0.18)	0.09 (−0.09 to 0.27)	−0.19+ (−0.41 to 0.02)	−0.36 (−0.87 to 0.14)
Discipline: Other (ref. physics)	−0.31 (−0.91 to 0.28)	−0.22 (−0.61 to 0.17)	−0.22 (−0.78 to 0.33)	−0.03 (−0.57 to 0.51)	0.01 (−0.31 to 0.32)	−0.65^**^ (−1.07 to −0.24)	−1.02^*^ (−1.83 to −0.22)
Position/status: postdoc (ref. postgraduate student)	0.14 (−0.08 to 0.36)	0.26 (−0.19 to 0.70)	0.28+ (−0.05 to 0.61)	0.15 (−0.19 to 0.49)	0.26^***^ (0.11–0.41)	0.41^**^ (0.15–0.68)	0.06 (−0.62 to 0.74)
Position/status: research scientist (ref postgraduate student)	0.29^*^ (0.01–0.57)	0.78^***^ (0.34–1.22)	0.82^***^ (0.38–1.27)	0.44+ (−0.00 to 0.89)	0.24 (−0.19 to 0.67)	−0.42 (−0.98 to 0.14)	0.41 (−0.45 to 1.28)
Position/status: junior faculty (ref. postgraduate student)	0.16 (−0.43 to 0.76)	0.40 (−0.19 to 0.99)	0.38 (−0.39 to 1.16)	0.50+ (−0.01 to 1.01)	0.19 (−0.28 to 0.66)	0.06 (−0.52 to 0.64)	1.21^*^ (0.18–2.25)
Position/status: mid-level faculty (ref. postgraduate student)	0.38 (−0.08 to 0.85)	1.06^**^ (0.42–1.71)	0.35 (−0.31 to 1.01)	0.60^***^ (0.26–0.95)	0.35^*^ (0.04–0.66)	0.15 (−0.41 to 0.70)	1.63^***^ (0.78–2.48)
Position/status: senior faculty (ref. postgraduate student)	0.41 (−0.14 to 0.96)	0.84^*^ (0.05–1.64)	0.54 (−0.20 to 1.28)	0.63^*^ (0.11–1.15)	0.22 (−0.21 to 0.65)	−0.12 (−0.79 to 0.55)	1.78^**^ (0.52–3.04)
Gender: women scientist (ref. men scientist)	−0.17 (−0.40 to 0.06)	−0.23^*^ (−0.41 to −0.05)	−0.39^***^ (−0.62 to −0.16)	−0.12 (−0.27 to 0.04)	0.04 (−0.17 to 0.25)	−0.00 (−0.26 to 0.26)	−0.18 (−0.53 to 0.16)
Age^a^	0.02^*^ (0.00–0.04)	−0.01 (−0.02 to 0.01)	0.03^**^ (0.01–0.06)	0.03^***^ (0.01–0.05)	0.01 (−0.00 to 0.03)	0.02+ (−0.00 to 0.04)	0.00 (−0.03 to 0.03)
Number of children^a^	−0.02 (−0.15 to 0.11)	−0.04 (−0.18 to 0.10)	0.10 (−0.08 to 0.28)	0.07 (−0.04 to 0.18)	0.02 (−0.13 to 0.17)	0.23^**^ (0.06–0.39)	−0.25 (−0.58 to 0.07)
Survey wave: May–June 2021 (ref. August–October 2021)	0.05 (−0.23 to 0.33)	−0.09 (−0.26 to 0.09)	−0.03 (−0.28 to 0.22)	−0.09 (−0.41 to 0.23)	−0.04 (−0.17 to 0.10)	−0.08 (−0.46 to 0.30)	0.26 (−0.14 to 0.66)
Observations	3,061	3,061	3,061	3,061	3,061	3,061	3,061
R-squared	0.10	0.05	0.16	0.21	0.10	0.06	0.09

Work and Well-Being in Science: An International Study (2021). *B*, unstandardized regression coefficients; CI, confidence interval. The statistics are fully survey-weighted. OLS regression of human flourishing as measured through the Flourishing Index.

^a^Treated as continuous; otherwise, the variables are categorical or binary.

^***^p < 0.001, ^**^p < 0.01, ^*^p < 0.05, ^+^*p* < 0.1.

## Discussion

Despite the concerns about the prevalence of mental health problems among early-career scientists (Bleasdale, 2019) and concerns about detrimental consequences of the COVID-19 pandemic (Chan et al., 2020), this cross-nationally representative study finds that on the whole, scientists in the disciplines of biology and physics seem to be flourishing moderately well, especially if they have a mid-level or senior faculty position.

Motivated by literature on the important role of aesthetics in science (McAllister, 1996; MacArthur, 2021), our work clearly establishes that the frequency of aesthetic experiences in the scientific workplace is positively, robustly, and strongly correlated with overall flourishing (Hypothesis 1). The size of this association is very large, especially when comparing scientists who never experience aesthetics in their scientific work compared to scientists who experience aesthetics on all measured dimensions at least weekly or more often. Demographic and other differences in terms of country, discipline, academic position, gender, age, and number of children between respondents have been held constant when assessing the association of aesthetics with flourishing. The findings imply that scientists in the disciplines of biology and physics who experience beauty, awe, and wonder in their work have higher flourishing in their lives.

One of the aims of positive psychology is to identify factors that lead to flourishing lives as opposed to only considering risk factors of negative mental health (Seligman, 2002). Our findings present a strong case for aesthetic experiences as a heretofore underexplored predictor of flourishing in a profession that has been described as plagued by growing mental health problems. In line with theories of intrinsic motivation, self-actualization, and self-determination (Deci and Ryan, 1985; Deci et al., 1994; Rawolle et al., 2016; Heintzelman, 2018; Thrash et al., 2019), aesthetic experiences appear to be a source of flourishing in science in the disciplines of biology and physics. Generally, the present study affirms findings from the literature that reported positive links between aesthetics and well-being (Mastandrea et al., 2019). Given the large size of this association, one could speculate that a lack of aesthetic experiences over a long period of time could play a role in scientists leaving academia or being more prone to experiencing burnout and mental health problems.

We posit that aesthetic experiences are an important source of intrinsic motivation and positive emotions in scientific inquiry; operating especially *via* the behavioral tendency towards novelty, challenge, and exploration, all of which support human flourishing (Heintzelman, 2018). Using the Multi-Motive Grid (Sokolowski et al., 2000), Rawolle et al. (2016) have shown that a lack of intrinsic motivation can amplify negative work outcomes (e.g., burnout), highlighting for us the importance of aesthetic experiences as a source of intrinsic motivation. This relationship also raises additional questions about the role of self-transcendent experiences and their neurophysiological underpinnings (e.g., the neuroaesthetic triad) as both motivational impetus and epistemological goal in science for future research to examine (Chatterjee and Vartanian, 2016; Thrash, 2021).

The summative scale of aesthetics provides a broad and exploratory assessment that roughly relates to three aesthetic components of beauty, awe, and wonder (McAllister, 1996; Dawkins, 2000; Wilczek, 2016; Gilbert, 2018; Gottlieb et al., 2018; MacArthur, 2021). Some of this might be due to the specifics of scientists’ research fields (e.g., an astrophysicist who works on computer models to create photorealistic images of stellar processes in the universe), but other aspects may also relate to lab cultures, discussions with colleagues, or science communication in which aesthetic experiences are openly considered or even fostered. Some work settings might provide more avenues for aesthetic encounters while other environments might provide fewer. In this sense, aesthetic experiences are not merely subjective or internal to the scientist but partly fostered externally in organizational and institutional cultures.

The associations of aesthetics with flourishing persist even net of negative workplace and life circumstances (Hypothesis 2). We found that the emotional exhaustion component of burnout and mistreatment in science are negatively associated with flourishing (Bleasdale, 2019), but those factors cannot explain away the positive association of aesthetics with flourishing, suggesting that in some cases, scientists may be able to protect their levels of flourishing despite workplace stresses if they have opportunities to encounter aesthetics in their work. While more work on the causal pathways is needed in future research, our findings could help identify a potential mechanism for the effect of aesthetics on flourishing that goes beyond correlates to point to a buffering or mediating role of such experiences in light of challenging and stressful work environments. Sensitivity analyses did not show evidence of flooring or ceiling effects for the frequency of aesthetic experiences measure, pointing to a linear relationship in which more frequent encounters of aesthetics in scientific work are incrementally better.

Even with the inclusion of serious psychological distress and its large effect on flourishing, the positive coefficient for the frequency of aesthetic experience in scientific work remains robust and large. This finding suggests that aesthetic experiences mainly operate independently on flourishing and that less than half of the association of aesthetic experiences with flourishing is explained *via* work stress outcomes like burnout, the COVID-19 control variables and general conditions like serious psychological distress. Similarly, aesthetic experiences themselves do not merely appear to act as opposites of psychological distress: Scientists in the disciplines that were part of this study can have aesthetic experiences (beauty, awe, and wonder) in their work despite challenging work and life circumstances and even when they suffer from serious psychological distress. This corresponds to insights on how self-actualization and transcendence can be achieved in the presence of challenging problems or despite a predisposition to sadness (Kaufman, 2021). Indeed, certain forms of mental illness can coexist with aesthetic experiences and there is debate that it might sometimes stimulate high artistic creativity (Cain, 2022). Aesthetic expression could promote flourishing on some domains, e.g., through a sense of purpose or meaning, even when other aspects of mental health are diminished.

Eudaimonia has increasingly been a focal aspect of well-being research (Ryff, 2018), and aesthetics may also shed light on understanding it better. Building on the above findings to address this question in Hypothesis 3, we found that aesthetic experiences in scientific work have the strongest associations with the meaning in life domain; then with character as relating to eudaimonic motivations or orientations; slightly weaker links with life satisfaction, mental health, and close social relationships; and no significant relationships with physical or financial stability. The fact that aesthetics play no role for physical health or financial stability is consistent with expectations about those associations but also demonstrates that scientists’ aesthetic experiences such as awe do not have a universal link with all domains of eudaimonic flourishing. Rather as expected from the literature, such links are strongest for the self-transcendent aspects of well-being (Chirico and Yaden, 2018), pointing the way for future investigations.

In this study we have measured aesthetic experiences that are intrinsic to scientific practice, and it seems that scientific work overall is a strong source of intrinsic motivation that allows scientists in the two studied disciplines to persist in the face of negatives that they experience both in science and in general (Ryan and Deci, 2001; Heintzelman, 2018). Accordingly, we believe that the removal of those unwanted pressures, forms of mistreatment, and unhealthy competition would improve the impact of the aesthetic experiences intrinsic to science as well as scientists’ overall flourishing. Some of these negatives have already been identified as targets for institutional reform (Ålund et al., 2020), which could also improve intrinsic motivation. Similarly, there has been evidence of the presence of broaden-and-build effects at both individual and institutional levels (Fredrickson, 2000), and organizations such as research universities could consider deepening scientists’ understanding and awareness of the beneficial role of aesthetics in their work.

To the best of our knowledge, this study is the first to examine the impact of scientists’ aesthetic experiences. Moreover, the large international sample coupled with the complex survey weights is representative of scientists in the disciplines of biology and physics in PhD granting institutions in the four countries and overcomes the limitations of other data that typically only have small convenience samples or high nonresponse bias. The study also employed multiple measures of well-being, primarily the multi-dimensional flourishing index that was developed by the Human Flourishing Program at Harvard University based on a comprehensive assessment of the well-being literature (VanderWeele et al., 2020). The flourishing scale is gaining in popularity and usage, and future studies would be able to compare their respective populations of interest to ours. Likewise, we included a separate measure of serious psychological distress (K6 scale; Kessler et al., 2002) as well as a key dimension of burnout, which allowed us to distinguish negative and positive domains of mental health (Westerhof and Keyes, 2010). The survey also included control variables for all the main factors of importance that might otherwise have confounded the analyses. The study also contributed to the field of positive psychology by having identified the positive role of aesthetics for flourishing.

In terms of limitations, the study employed a cross-sectional research design and is therefore unable to ascertain causality between aesthetic experiences and flourishing. It is possible that reverse causal processes are taking place (e.g., lower well-being diminishes ability to experience aesthetic aspects of work), and one might also suggest that to some extent, aesthetics are a component rather than predictor of flourishing. Other limitations apply to some of our measures. The burnout measure only captures the aspect of emotional exhaustion and not the other two main components of personal efficacy and cynicism (Maslach et al., 1997). The mistreatment variable captured a variety of different mistreatment types, but the intensity or frequency of such mistreatment were missing.

While our novel aesthetics measure is based on consultations with experts in the field and closely related findings from the relevant literature, it has not yet been validated. This study grouped the three components of aesthetics (beauty, awe, and wonder) together, but a finer differentiation to increase specificity and measurement would be useful (e.g., awe might be better measured independently on an intensity scale instead of a frequency scale). Other components of aesthetics such as inspiration and creativity could also be missing from the present specification (Wright and Pascoe, 2015; Cui et al., 2020; Thrash, 2021). Aesthetic experiences may be linked with religious or spiritual predispositions (e.g., religious scientists are more likely to be attuned to wonder and awe; Viladesau, 2014), but the limited scope of this paper does not allow for the parsing of these nuances. This study focused on work-related aesthetic experiences that are particular to an occupation/community; there may be other types of aesthetic experiences, which have not been considered, that could affect dimensions of flourishing differently (e.g., scientists’ consumption of art or music), and the aesthetic dimensions measured here may not be relevant to other occupations.

Future research should consider aesthetic experiences as a potentially key predictor of flourishing, especially for the eudaimonic dimensions of meaning. The association of aesthetics with flourishing is strong among scientists in the disciplines of biology and physics (during the COVID-19 pandemic), but aesthetic experiences could also be important in other settings or over different time frames, for instance in education (Adu-Agyem and Enti, 2009), and contextualized by occupational identity. The present study and its original aesthetic frequency scale (see Figure 1) may act as a useful reference point for other researchers to create comparable indices of aesthetic experiences (e.g., for other scientific disciplines, other professions or work settings, or even to develop aesthetics measures that are applicable to the general population). As the association is robust cross-nationally, it is likely that aesthetics experiences play a prominent role for human flourishing in different cultures. One might even consider interventions to increase appreciation of aesthetic factors in various workplace or other settings, as part of mindfulness sessions, psychological coaching, or psychotherapy (Knill, 1995) for instance. It will be worthwhile to investigate the association of aesthetics with related concepts, such as optimism, purpose in life, psychological strengths, and resilience (Kashdan et al., 2021), and to replicate the present findings with other flourishing scales. It is also worthwhile to differentiate certain conceptual aspects, such as mathematical beauty (Zeki et al., 2014) that might resonate more strongly with physicists, from other visual aspects such as symmetry that might resonate more strongly with biologists, and from aspects that span the two such as elegance. Finally, future research should also examine the specific content, modality, and neuropsychological underpinnings of aesthetic experiences in science (Varga, 2021).

## Conclusion

This international study has established a very strong and positive association between the frequency of aesthetic experiences in scientific work and higher levels of flourishing among biologists and physicists in India, Italy, the United Kingdom, and the United States. Aesthetic experiences in scientific work have a robust and independent association with flourishing even when controlling for various negative life circumstances such as workplace stressors (burnout, job/publication pressure, and mistreatment experiences in the scientists’ careers) and taking into account general stressors (COVID-19 impacts, other stressful events, serious psychological distress, chronic health conditions). These findings suggest that aesthetic experiences in scientific work in the two studied disciplines have a unique pathway with flourishing—possibly reflecting self-transcendent well-being and in line with positive psychological theories of intrinsic motivation, self-actualization, and flow—that is mostly operating independently of other sources of stress at work and in life in general. Indeed, the association of aesthetic experience is strongest with the eudaimonic subdomain of meaning from the flourishing index. While a causal interpretation of the reported associations would require experimental or longitudinal validation, the results suggest research group leaders and university managers in general should consider fostering scientific workplace environments that improve the opportunities for experiencing the aesthetic dimensions of science or that allow for the sharing and discussion of aesthetic experiences and positive emotions more generally.

## Data availability statement

The raw data supporting the conclusions of this article will be made available by the authors, without undue reservation.

## Author contributions

CJ conducted all statistical analyses and contributed to the study design and the paper writing. PV contributed to the study design, the literature review and paper writing. BV contributed to the study design, data collection and advised on the statistical analyses and writing. All authors contributed to the article and approved the submitted version.

## Funding

Data collection for this paper was funded by a grant from the Templeton Religion Trust (TRT0296, PI: BV).

## Conflict of interest

The authors declare that the research was conducted in the absence of any commercial or financial relationships that could be construed as a potential conflict of interest.

## Publisher’s note

All claims expressed in this article are solely those of the authors and do not necessarily represent those of their affiliated organizations, or those of the publisher, the editors and the reviewers. Any product that may be evaluated in this article, or claim that may be made by its manufacturer, is not guaranteed or endorsed by the publisher.

## Footnotes

^1^https://covid19.who.int (accessed on 04/18/2022).

^2^It was not necessary to apply listwise deletion when respondents (in 30 cases) had partially missing values on some but not all of the mistreatment items; this simply resulted in a lower but still valid count of mistreatment experiences.

^3^In the full flourishing index, the physical and mental health items are grouped together under one domain, but here we separated them as distinct domains.
